# The trade‐off between fecundity and egg size in a polymorphic population of Arctic charr (*Salvelinus alpinus* (L.)) in Skogsfjordvatn, subarctic Norway

**DOI:** 10.1002/ece3.2669

**Published:** 2017-02-26

**Authors:** Aslak Smalås, Per‐Arne Amundsen, Rune Knudsen

**Affiliations:** ^1^ Department of Arctic and Marine BiologyUiT—the Arctic University of NorwayTromsøNorway

**Keywords:** life history strategies, reproductive investment, salmonids, trade‐offs

## Abstract

Reproductive traits differ between intralacustrine Arctic charr morphs. Here, we examine three sympatric lacustrine Arctic charr morphs with respect to fecundity, egg size and spawning time/site to assess reproductive investments and trade‐offs, and possible fitness consequences. The littoral omnivore morph (LO‐morph) utilizes the upper water for feeding and reproduction and spawn early in October. The large profundal piscivore morph (PP‐morph) and the small profundal benthivore morph (PB‐morph) utilize the profundal habitat for feeding and reproduction and spawn in December and November, respectively. Females from all morphs were sampled for fecundity and egg‐size analysis. There were large differences between the morphs. The PB‐morph had the lowest fecundity (mean = 45, *SD* = 13) and smallest egg size (mean = 3.2 mm, *SD* = 0.32 mm). In contrast, the PP‐morph had the highest fecundity (mean = 859.5, *SD* = 462) and the largest egg size (mean = 4.5 mm, *SD* = 0.46 mm), whereas the LO‐morph had intermediate fecundity (mean = 580, *SD* = 225) and egg size (mean = 4.3, *SD* = 0.24 mm). Fecundity increased with increasing body size within each morph. This was not the case for egg size, which was independent of body sizes within morph. Different adaptations to feeding and habitat utilization have apparently led to a difference in the trade‐off between fecundity and egg size among the three different morphs.

## 1 Introduction

In studies of life history evolution, consideration of traits related to reproduction is important because of their role in determining individual fitness (Stearns, 1992). Limiting resources and developmental constraints lead to trade‐offs, such as between growth and reproduction, between survival and reproduction, and between quantity and quality of offspring (Roff, 1992). Diverging selection pressure between individuals could lead to different trade‐off regimes, thus leading to dissimilarities in life history traits. Fish reproductive effort can be measured as the egg mass per female, which is the product of fecundity and egg size (Duarte & Alcaraz, 1989). Egg size and fecundity could be different between individuals with similar egg mass. Dissimilar egg size and egg numbers between individuals could arise from genetic differences, environmental factors or a combination (Wootton, 1998). Energy availability and/or female size are factors that constrain both egg number and egg size, as larger eggs only can be produced at the cost of reduced fecundity (Elgar, 1990; Wootton, 1998). Thus, the trade‐off between egg size and fecundity is important for determining individual fitness.

The optimal egg size that maximizes fitness for a given female is the egg size at which the product of juvenile survival and fecundity is at a maximum (Sibly & Calow, 1986). Smith & Fretwell's (1974) model of the trade‐off between fecundity and egg size states that fecundity and not egg size varies with the amount of acquired resources. Thus, each population should have an “optimal” egg size. In several fish species, such as the Atlantic salmon (*Salmo salar*), dace (*Leuscicus leuscicus*) and coho salmon (*Oncorhynchus kisutch*), both fecundity and egg size increase with female body size (van den Berghe & Gross, 1984; Mann & Mills, 1985; Thorpe, Miles, & Keay, 1984). This means that for salmonids, larger individuals, which may have higher absolute fecundity compared with smaller individuals, invest more energy into each offspring thus reducing relative fecundity (number of eggs per unit body weight) compared with smaller individuals. Additional model improvements have further enlightened our understanding of the relationship between fecundity and egg size, also for fishes with large phenotypic variation, and in which egg size correlates with body size (Hendry, Day, & Cooper, 2001; Sargent, Taylor, & Gross, 1987). Epigenetic effects, like maternal investment in egg size, have been suggested to be an adaptation to fluctuating environments to increase fitness (Leblanc, Benhaim, Hansen, Kristjansson, & Skulason, 2011). Maternal investment in egg size could also be important in deciding offspring size at hatching, offspring behavior and early growth, food intake rate, and resource use Leblanc, Kristjánsson, & Skúlason, 2014, thus deciding behavior and body size of offspring through the early ontogeny.

Arctic charr (*Salvelinus alpinus*) is a highly polymorphic species, which often may occur as two to four sympatric morphs within the same lake (Klemetsen, 2010; Klemetsen et al., 2003). In Arctic charr, egg size is highly variable (Klemetsen, 2010; Klemetsen et al., 2003; Wallace & Aasjord, 1984). Leblanc et al. (2011) showed that differences between juveniles in behavior and mobility was related to egg size and thus could play a central role in the divergence process and maintenance of polymorphisms in Arctic charr. Smaller morphs of Arctic charr produce fewer and smaller eggs compared with their larger morph pair (Klemetsen et al., 2003). This is true for the profundal morph in Fjellfrøsvatn, Norway, and the small benthic morph in Thingvallavatn, Iceland (Eiríksson, Skulason, & Snorrason, 1999; Klemetsen, Elliott, Knudsen, & Sørensen, 2002; Klemetsen et al., 2003; Sandlund et al., 1992). Arctic charr populations and morphs also show large variations in other traits like morphology, behavior, and other life history parameters.

In Skogsfjordvatn, northern Norway, three different morphs of Arctic charr have been described (Skoglund, Siwertsson, Amundsen, & Knudsen, 2015). One of these morphs predominantly utilizes the littoral zone, whereas the other two utilize the profundal. Smalås, Amundsen, and Knudsen (2013) found great variations in life history traits such as somatic growth and age and size at maturity between the three morphs. The profundal spawning, benthivore morph (PB‐morph) is small and matures at the youngest age and smallest body size compared with the two other morphs. The littoral spawning, omnivore morph (LO‐morph) and the profundal spawning, piscivore morph (PP‐morph) grow to similar body sizes, but differ in respect to age and length at maturity, with the PP‐morph being distinctly larger and older at maturity than the LO‐morph (Smalås et al., 2013). A few individuals belonging to the LO‐morph population exhibit an anadromous migratory behavior; however, the number of anadromous individuals is negligibly small (Siwertsson, Refsnes, Frainer, Amundsen, & Knudsen, 2016). The two profundal morphs differentiate in resource use as one feeds on small benthic invertebrates and the other is mainly piscivorous (Knudsen, Amundsen et al., 2016; Knudsen, Gjelland, et al., 2016; Skoglund et al., 2015; Smalås et al., 2013). The piscivorous form feeds heavily on all charr morphs, also including the three‐spined stickleback (*Gasterosteus aculeatus* L.) in its diet (Knudsen, Gjelland, et al., 2016). The LO‐morph individuals utilize both benthic food resources in shallow‐water areas and zooplankton in open water habitats (Skoglund et al., 2015). Here, we examine the three Arctic charr morphs in respect to fecundity, egg size, and time and place of spawning, addressing variations both between and within morphs. We also address possible fitness consequences of the observed variations. We hypothesize that the smallest morph (PB‐morph) has the lowest absolute fecundity and the smallest egg size. The opposite was expected for the largest morph (PP‐morph), which we hypothesize to have the highest absolute fecundity and largest egg size. The LO‐morph adult body size is only slightly smaller compared with the PP‐morph, thus expecting similar absolute fecundity and egg sizes as the PP‐morph. The size difference between adult females from the PB‐morph and the PP‐morph should lead to difference in absolute fecundity (Smith & Fretwell, 1974) and also a difference in egg size as observed for most salmonids (Thorpe et al., 1984), despite experiencing similar abiotic conditions (i.e., temperature, light, hydrostatic pressure). We hypothesis that time and place of spawning are different between the two deep‐living morphs and the LO‐morph. Difference in spawning time and spawning place between shallow‐water morphs and deep‐living morphs are experienced in other systems with similar charr morph populations. In Fjellfrøsvatn northern Norway, the profundal morph spawn in February in the profundal zone, while the littoral morph spawn in September in the littoral zone (Klemetsen, Amundsen, & Hermansen, 1997).

## 2 Material and Methods

Lake Skogsfjordvatn (surface area 13.6 km^2^, maximum depth 100 m, 20 m above sea level) is located on the island Ringvassøya, Troms county, northern Norway (69°55′48′′N, 19°9′36′′E). Alpine landscape and birch forests dominate the drainage area. The lake is dimictic and oligotrophic, has low alkalinity and is moderately affected by humic influx, and is usually ice‐covered from December to early June. The lake is exposed to the midnight sun 2 months during summer and near total darkness during the polar night 2 months in the winter. The lake connects to the sea via a 1 km long river, and the fish community in the lake consists of anadromous and resident Arctic charr, brown trout (*Salmo trutta* L.), and Atlantic salmon, along with three‐spined stickleback (*Gasterosteus aculeatus* L.) and European eel (*Anguilla anguilla* L.).

Fish sampling was done over 2–3 days each month from May 2011 until January 2012. Multimesh gillnets, ranging from 10 to 45 mm knot to knot, were used in the benthic areas (littoral, 0–15 m, and profundal, >20 m) and in the pelagic zone. The charr were identified to morph by their external appearance. The littoral LO‐morph has a relatively small head, red to orange abdominal spawning colors, and a silvery background body color. The PB‐morph is small‐sized, has bands (par marks) along the side of the body, thus having a paedomorphic appearance, and has a relatively blunt and rounded head. The PP‐morph has a relatively large and elongated head, a dark gray or black body color with white spots, a relatively slim and elongated body shape, and sharp teeth on the tongue and the palate (for more details and description of the morphs, see Skoglund et al., 2015). The anadromous charr resembles the LO‐morph; however, it has adapted a more pelagic camouflage and thus has a more silvery body color than the LO‐morph. Genetic studies have concluded that it is a part of the LO‐morph population, and they have common breeding time and spawning sites (R. Knudsen, unpublished data).

Fish were weighed to the nearest gram, fork length (length from the tip of the nose to the shortest rays on the caudal fin) was measured to the nearest millimeter, and otoliths were removed for age determination. The sex and stage of sexual maturity of each fish was decided by examining the gonads. Ovaries of sexual mature female fish were collected in the field from September through January and were weighed and preserved and stored in Gilson`s fluid (100 ml 60% alcohol, 800 ml water, 15 ml 80% nitric acid, 18 ml glacial acetic acid, 20 g mercuric chloride) until analyzed (Holden & Raitt, 1974). Friedland et al. (2005) found that Gilson fluid had a significantly effect on egg size after 161 days of storage; however, they suggested that in the two‐first months of storage no effect on oocyte diameter was detectable. Egg size measurements were carried out a few weeks (2–3) after sampling for all fish to avoid the effect Gilson fluid might have on oocyte diameter. The total number of eggs (i.e., clutch size) for each individual was estimated gravimetrically. Two subsamples of about 1 g of eggs was counted for each fish and fecundity was calculated as: *F* = nG/g where *F* is fecundity, *n* is the number of eggs in the subsample, G is the total weight of the ovaries and g is the weight of the subsample. The average of the two subsamples was calculated (Holden & Raitt, 1974).

Fifteen oocytes from each fish were taken at random and measured separately to the nearest 0.01 mm using a dissecting microscope. An average egg size for each female fish was then calculated. All female fish analyzed for egg size measurements were sampled close to their respective spawning time. Thus, only female fish with fully ripe gonads were included. The difficulty of catching a large number of females with fully ripe gonads was high; thus, the sampling size off all three morphs is relatively low. The within‐morph variation in body size and age in the analyzed data is as follows: LO‐morph = length from 213 to 300 mm and age from 4 to 9 years, PB‐morph = length from 93 to 128 mm and age from 3 to 6 years and PP‐morph = length from 280 to 403 mm and age from 8 to 12 years. To determine time and place of spawning, the presence/absence of ovulating females and males with running gonads was identified in the gillnet catch for all sampling sites and periods.

### 2.1 Statistical analyses

All statistical analyses were carried out using R 2.15.1 software. Two‐way‐ANOVA and stepwise regression were used to determine the factors to be included in the models to explain the response variables egg size and fecundity. Both response variables (egg size and fecundity) were normally distributed. Regression analyses were used to test for linear relationships between the response variables and the continuous explanatory variables (weight and length), with different levels for the factorial explanatory variable of morph (three levels). A post hoc test (Tukey HSD) was used for testing for differences in mean fecundity and mean egg size between morphs. A generalized linear model (GLM) was used to calculate average Julian day of spawning, with the proportion of spawning individuals as the response variable and Julian day as the predictor variable for all three morphs. A chi‐square test, on residual deviance and degrees of freedom, was conducted to assess model fitness. For all three morphs, the null hypothesis could not be rejected, thus confirming that the fitted values were not significantly different from the observed (LO‐morph: *p* = .999, PB‐morph: *p* = .999, PP‐morph: *p* = .998).

## 3 Results

### 3.1 Egg size

**Figure 1:**
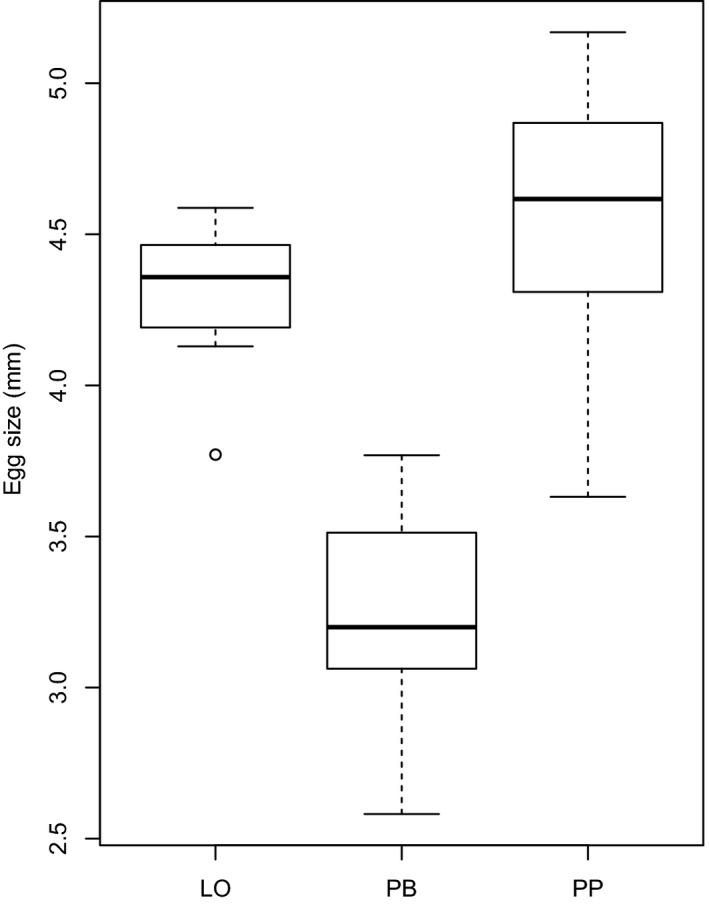
The egg size in diameter for the PB‐morph (*n* = 19), LO‐morph (*n* = 11) and PP‐morph (*n* = 12) of Arctic charr in Skogsfjordvatn. The box‐whisker plots show the median (thick line), the 50% interquartile (box), values that are up to 1.5 times different from the interquartile (whiskers) and outliers (circles)

There were significant differences in egg size between morphs (ANOVA: *F*‐value (2, 39) = 62.92, *p* < .0001). A post hoc test (Tukey HSD) revealed a significant difference in mean egg size (*q*‐value (2, 39) = 3.445) between the PB‐ and LO‐morph (*p* < .0001), and between the PB‐ and PP‐morph (*p* < .001), but not between the LO‐ and PP‐morph (*p* = .29). The egg size of the PB‐morph was thus smaller than for the other two morphs (Table 1, Figure 1). However, egg size was not significantly affected by body weight within the Arctic charr morphs in Skogsfjordvatn (ANCOVA: *F*‐value (1, 38) = 1.2512, *p* = .2702). This was supported by a linear model for each morph with egg size as the response variable and weight as the predictor variable. There was no effect on egg size from body weight for all three morphs, the LO‐morph (*t*‐value (1, 9) = 0.70, *p* = .502), the PB‐morph (*T*‐value (1, 17) = 1.586, *p* = .131), and the PP‐morph (*t*‐value (1, 10) = 0.741, *p* = .476). This confirms that egg size remained relatively constant for all body sizes within each of the different morphs.

**Table 1:** Mean fecundity (±*SD*), mean relative fecundity (±*SD*), and mean egg size (±*SD*) for the three arctic charr morphs in Skogsfjordvatn

Morph	*n*	Mean fecundity (*SD*)	Mean relative fecundity (Eggs/kg [*SD*])	Mean egg size (*SD*)
LO	11	580.1 (225)	2934 (447)	4.31 (0.23)
PB	19	45.3 (13)	3927 (854)	3.22 (0.32)
PP	12	859.5 (462)	2510 (523)	4.54 (0.46)

### 3.2 Fecundity

**Figure 2:**
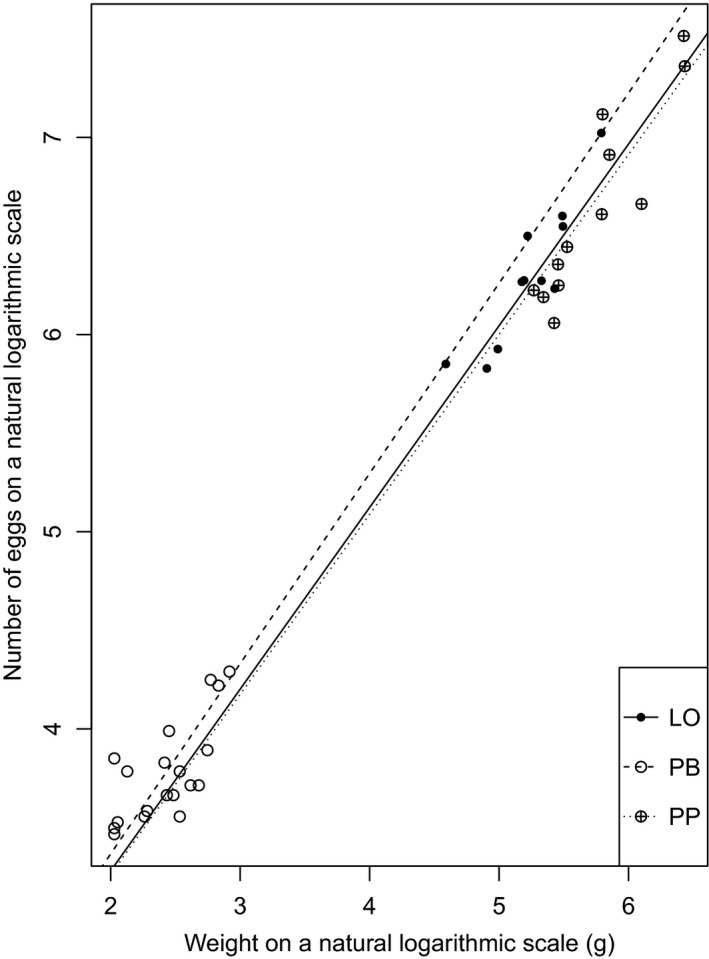
Relationship between body weight (g) and fecundity for the three different Arctic charr morphs in Skogsfjordvatn on a log‐log scale. The regression lines explain the main effect of body weight and morph on fecundity; in addition, there is an interaction effect of egg size on the slope for all three morphs

Mean fecundity was significantly different between the three morphs (ANOVA: *F*‐value (2,39) = 36.037, *p* < .0001). A post hoc test (Tukey HSD) revealed a significant difference in mean fecundity (*q*‐value (2, 39) = 3.445) between the PB‐ and LO‐morph (*p* < .0001), between the PB‐ and PP‐morph (*p* < .001), and between the LO‐ and PP‐morph (*p* = .045). The PB‐morph had the lowest observed fecundity and the PP‐morph had the highest, whereas the exact opposite was evident for relative fecundity, where the PB‐morph had the highest and the PP‐morph had the lowest values (Table 3). Log‐log‐transformed regression analyses (*F*‐statistic (2, 39) = 1174, adjusted *R* ^2^ = 0.983, *p* < .0001) revealed a significant increase in fecundity with increasing body weight (*t*‐value (2, 39) = 10.859, *p* < .0001), and in addition, there was an interaction effect of egg size (*t*‐value (2, 39) = −2.168, *p* = .0363) on the slope with a relative increase in fecundity with decreasing egg size (Figure 2).

### 3.3 Time and place of spawning

The estimated spawning times revealed distinct differences between the Arctic charr morphs in Skogsfjordvatn. The individuals from the LO‐morph spawned from late September to early October, with an estimated average spawning day of October 7 (Julian day = 280) (GLM: *t*‐value (8,7) = 64.84, *p* < .001). The majority of the individuals from the PB‐morph spawned from late November to early December, with an estimated average spawning day of November 26 (Julian day = 330) (GLM: *t*‐value (8,7) = 19.30, *p* < .001). The PP‐morph population had a broader spawning period than the other two morphs, as some individuals were spent already in October, while the main spawning period was in the middle of December, with an estimated average spawning day of December 14 (Julian day = 348) (GLM: *t*‐value (8,7) = 3.302, *p* = .0108). Ovulating females were only found in the littoral zone for the LO‐morph, whereas ovulating females from the PP‐morph and PB‐morph in contrast only were found in the profundal zone.

## 4 Discussion

Reproductive traits differed between the three Arctic charr morphs in Skogsfjordvatn. The profundal, small‐sized PB‐morph had significantly lower fecundity and smaller eggs than the two other morphs. Furthermore, the PP‐morph had higher fecundity, but not larger eggs than the similar‐sized LO‐morph. Fecundity increased with increasing body weight within all morphs, whereas we found no effect of body weight on egg size within the morphs. Mean relative fecundity decreased from the smallest‐sized morph (PB‐morph) to the largest‐sized morph (PP‐morph). The spawning time was relatively similar for the two deep‐water morphs that experience identical environmental conditions (temperature, hydrostatic pressure, and light regimes). The spawning timing for the PB‐ and PP‐morphs was, however, different from the littoral‐spawning LO‐morph, which had a similar spawning time as observed from littoral‐spawning charr in other nearby lake systems (Klemetsen et al., 1997).

The body size differences between the small‐sized PB‐ and the large‐growing LO‐ and PP‐morphs are also reflected in the number of eggs produced. The results suggest that the trade‐off trajectories between number and size of eggs (and thus offspring) act differently between the morphs. The smaller‐sized PB‐morphs seem to invest less energy in each offspring (smaller eggs) in order to produce a higher number of offspring compared with the other two morphs. This was also a pattern found in Arctic charr morphs in both Fjellfrøsvatn and Thingvallavatn, where smaller‐sized morphs produced smaller eggs compared with sympatric living, larger‐sized morphs (Eiríksson et al., 1999; Klemetsen et al., 2002, 2003; Sandlund et al., 1992). Hence, all corresponding sympatric morph pairs in Fjellfrøsvatn, Thingvallavatn, and the Skogsfjordvatn Arctic charr morphs seem to have similar trade‐off regimes associated with fecundity and egg size. Additionally, the benthic food habitat that these morphs utilize provides less free energy for gonad development compared with the diet of their respective morph pair. Thereby, when absolute fecundity is low (i.e., the PB‐morph), females may increase their relative fecundity by investing less in each offspring by producing smaller eggs. Thus, natural selection may facilitate a smaller egg‐size optima to increase fitness. Vice versa, when absolute fecundity is high (i.e., the LO‐ and PP‐morphs), the relative fecundity decreases as more energy is invested in each offspring by having larger egg sizes. Thereby, natural selection may promote a larger egg‐size optima to increase their fitness.

The egg size of the PB‐morph in Skogsfjordvatn is small compared with Arctic charr populations found elsewhere (Klemetsen, 2010). As discussed earlier, most of the explanation might come from the situation that populations consisting of smaller‐sized females produce eggs that are smaller compared with populations consisting of larger‐sized females. However, the eggs produced by females from the PB‐morph are extremely small and are to our knowledge the smallest ever reported for Arctic charr (Klemetsen, 2010; Klemetsen et al., 2003). The profundal morph in Fjellfrøsvatn has very similar life history traits as the PB‐morph in Skogsfjordvatn regarding age and size at maturity, growth trajectories, and longevity (Klemetsen, 2010; Smalås et al., 2013). The only life history traits that differ between the profundal morph in Fjellfrøsvatn and the PB‐morph in Skogsfjordvatn are in fact the egg size and fecundity, with average egg sizes of 3.8 and 3.2 mm and average fecundities of 30.4 and 45.7 eggs, respectively (Klemetsen et al., 2003). The most obvious difference between those two systems is that in Fjellfrøsvatn there is a lack of a specialized piscivore predator in the profundal zone (Knudsen, Amundsen et al., 2016), which may imply a different selection regime on life history traits between these systems. Generally, life history theory states that in systems with high predation pressure, the prey should invest more in reproduction early in life and produce more offspring at the expense of the quality of offsprings (Johnson & Belk, 2001; Walsh & Reznick, 2008). Hence, in Skogsfjordvatn, natural selection may have promoted an increased fecundity of the PB‐morph instead of decreasing the age and size of maturity compared with the profundal morph in Fjellfrøsvatn. The observed size (<7 cm) and age (~3 years) at maturity may in both lakes be at the lowest possible threshold for these deep‐water morphs. As a response to high predation pressure, evolution may have promoted decreasing egg size and thereby increasing fecundity of the PB‐morph in Skogsfjordvatn. Hence, the observed patterns may meet the assumptions from general life history theory in order to adapt the optimal life history strategy in the presence or absence of a predator.

The egg size did not change with increased female body size within each of the morphs in Skogsfjordvatn. These results lend support to the classic egg‐size optimization model of Smith and Fretwell (1974), which suggests that each population should have an optimum egg size. If selection optimizes investment per offspring, then offspring size may be more or less constant within populations (Morris, 1987; Smith & Fretwell, 1974). There should be a single optimal egg size and female fecundity should have low influence unless there exists a strong density‐dependent sibling competition (Parker & Begon, 1986). On the contrary for salmonids, the general assumptions are that both fecundity and egg size increase with increasing body size within populations (Einum & Fleming, 1999; Thorpe et al., 1984; Wootton, 1998). The increase in egg size by body size is suggested to be adaptive, with larger females obtaining a higher fitness by producing larger eggs (Fleming, 1996). This seems to be less obvious within the Arctic charr morphs in Skogsfjordvatn.

The ancestral population of charr in Skogsfjordvatn is assumed to be of an anadromous origin; thus, it is somewhat surprising to observe no effect of increased body size on egg size within morphs, as observed in other anadromous salmonid populations. However, most of these studies are from riverine spawning salmonids and may not apply for Skogsfjordvatn Arctic charr morphs. Riverine spawning salmonids experience large fluctuation in many environmental conditions (e.g., water level, flooding, ice, temperature) during their egg/hatchling/larval stage. Environmental variability over years may lead to less uniform production of egg sizes between females within the same population (Hutchings, 1991). Thereby, the optimal propagule size will vary between years and selection will thus not favor the same optimal size from one year to the next. In lakes in contrast, the environmental conditions (e.g., temperature, light, water level) are very stable and predictable between years, especially in the profundal zone. Furthermore, most studies conducted on maternal traits as egg size and fecundity in salmonids are from territorial riverine populations, especially Atlantic salmon, but also brown trout. These populations experience high intraspecific density‐dependent competition for food and space in early life stages (Einum & Fleming, 2000; Jonsson, Jonsson, Brodtkorb, & Ingebrigtsen, 2001; Louhi, Robertsen, Fleming, & Einum, 2015), where increased size at hatching may be decisive for the competitive success (Einum & Fleming, 2000). This may in less degree be the case for the present Arctic charr morphs, where density‐dependent intrapopulation competition may be weak, or at least weaker than competition among morphs. Hence, for the PB‐morph it might be more important to be a stronger competitor toward the PP‐morph hatchlings rather than toward their own conspecifics in order to increase their fitness by depriving the possibility of being predated on later in life.

Our studies of the two deep‐living Arctic charr morphs in Skogsfjordvatn suggest that these morphs experience very similar abiotic conditions throughout their ontogeny. In other words, there exist no obvious differences in abiotic factors that could explain the observed differences in body size between the two deep‐living charr forms at all life history stages, including also the egg stage. Leblanc, Kristjánsson, and Skúlason (2014) found in domesticated Arctic charr that larger‐sized eggs led to larger‐sized embryos that had more yolk and thus more available energy. Their findings suggested that embryos from larger eggs depleted their yolk reserves faster and had a higher growth rate than embryos from smaller eggs, but there was no difference in mortality rates corresponding to egg size (Leblanc et al., 2014). Other studies have concluded that charr originating from smaller eggs develop differently than charr originating from larger eggs (Eiríksson et al., 1999; Leblanc et al., 2011), with charr individuals from larger eggs/embryos investing more energy in somatic growth and those from smaller eggs/embryos investing more energy in bone and structural development (Eiríksson et al., 1999). Leblanc et al. (2014) also found that individual differences in egg size were positively correlated with differences in body size of the juveniles, suggesting that egg size could transfer into long‐term growth and behavioral differences. This could suggest that a difference in maternal investment in egg size between the two profundal morphs in Skogsfjordvatn leads to differences in growth trajectories, allocation of energy, yolk depletion, and behavior over a long period of time. Thereby, egg size may predecide final body size differences between the two profundal morphs in Skogsfjordvatn. This could suggest that egg‐size differentiation between the two morphs is important for the creation and maintenance of polymorphic charr populations.

The time and place of spawning was very different between the late autumn/early winter (during the polar night) spawning of the two deep‐water spawning PB‐ and PP‐morphs compared with the early autumn spawning on shallow water of the LO‐morph in Skogsfjordvatn. This pattern has also been supported by a clear difference in the seasonal development of the hormone status of the LO‐ and PP‐morphs (Hestdahl, 2013). This is similar to other morph pairs that segregate in deep water and shallow water (Klemetsen et al., 1997). We agree with Klemetsen et al. (1997) that suggested that the temperature difference between the two spawning habitats (littoral for the LO‐morph and profundal for the other two morphs) could explain the difference in breeding time between the shallow‐ and deep‐water spawning morphs. The temperature during winter stratification is higher in the profundal zone compared with the shallow water in the littoral zone (Klemetsen et al., 1997). This could lead to that the eggs that develop in shallow water need longer time to hatch than eggs that develop in the profundal zone; however, hatching time might be similar for all three morphs. The PB‐ and PP‐morphs spawned much closer both in respect to time and place, and crossbreeding may thus be more likely to happen between these two morphs. However, genetic analysis has revealed large genetic differences between the morphs (Knudsen, R. unpublished data), demonstrating that cross‐breeding likely not happen between the two deep‐water morphs or that the survival of their hybrids are low. The large differences in body size between the PB‐ and PP‐morphs may be an important factor for preventing or reducing any crossbreeding between the two morphs. Moreover, as the PP‐morph feeds heavily on small‐sized Arctic charr including also individuals from the PB‐morph (Knudsen, Gjelland, et al., 2016), these predator–prey interactions may strongly inhibit the use of common breeding sites. The two deep‐water living morphs therefore most likely segregate in their breeding sites.

The trade‐off regime between fecundity and egg size acts differently between the morphs in Skogsfjordvatn. In particular, the small‐sized PB‐morph reduces the egg size to produce a relatively higher number of offspring compared with the two other morphs. This egg‐size differentiation may be an important explanation for the contrasting life history strategies between the two deep‐water morphs, which the similarity in experienced abiotic conditions could not explain. Dissimilarity in egg size may lead to a great difference between the morphs already at the hatchling stage (size, growth rate, yolk reserves), and these difference may be sustained through ontogeny, thus, “evolutionary” maintain morph separation and segregation over time.

## Conflict of interest

None declared.
